# The Overexpression of SLC25A13 Predicts Poor Prognosis and Is Correlated with Immune Cell Infiltration in Patients with Skin Cutaneous Melanoma

**DOI:** 10.1155/2022/4091978

**Published:** 2022-05-14

**Authors:** Yue lv, Chun-hui Yuan, Lu-yao Han, Gao-ru Huang, Ling-ce Ju, Ling-hui Chen, Hai-ying Han, Chong Zhang, Ling-hui Zeng

**Affiliations:** ^1^Department of Nursing, School of Medicine, Zhejiang University City College, Hangzhou, Zhejiang, China 310015; ^2^Department of Pharmacology, School of Medicine, Zhejiang University City College, Hangzhou, Zhejiang, China 310015; ^3^Thyroid Surgery Department, the First Affiliated Hospital, School of Medicine, Zhejiang University, Hangzhou, China 310003

## Abstract

**Purpose:**

Skin cutaneous melanoma (SKCM) is one of the most malignant and aggressive cancers with poor prognosis due to its rapid progression towards metastasis. Thus, finding clinically relevant biomarkers for early diagnosis, prognosis, and therapy prediction is essential. This study focused on the identification of SLC25A13 as a novel biomarker for SKCM and is aimed at investigating the biological functions of solute carrier family 25 member 13 (SLC25A13) in the development of SKCM.

**Methods:**

GEPIA was used to analyze the diagnostic and prognostic values of SLC25A13 in SKCM using the TCGA dataset. PrognoScan was used to validate the prognostic value of SLC25A13 and its coexpressed genes in SKCM. TISIDB was established to reveal the relationship between the expression of SLC25A13 and immune infiltration in SKCM. The protein expression of SLC25A13 in SKCM was evaluated by the Human Protein Atlas. The signaling pathways and biological functions of SLC25A13 in SKCM were analyzed by LinkOmics. Metascape was applied to analyze the functional enrichment analysis of SLC25A13. Protein-protein interaction analysis of SLC25A13 was performed by GeneMANIA.

**Results:**

The mRNA and protein levels of SLC25A13 in the SKCM were much higher than those in the normal tissue. Furthermore, the overexpression of SLC25A13 predicts worse outcomes of SKCM patients. Moreover, the SLC25A13 expression was negatively correlated with the immune infiltration level of SKCM. The overexpression of SLC25A13 coexpressed genes, such as ACLY and AFG3L2, and SCL25A13 interacting genes also predicted the unfavorable prognosis of SKCM patients. Gene Ontology (GO) and Kyoto Encyclopedia of Genes and Genomes (KEGG) analysis of SLC25A13 coexpressed genes showed that these genes are enriched in ATPase activity, cell cycle, mTOR, and VEGFA-VEGFR2 signaling pathways, which were relevant to tumor development and angiogenesis. Gene set enrichment analysis (GSEA) demonstrated that the SLC25A13 expression was related to infiltrating immune cells in SKCM.

**Conclusion:**

Our findings revealed that SLC25A13 might be a potential prognostic and therapeutic biomarker for SKCM.

## 1. Introduction

The incidence of skin cutaneous melanoma (SKCM) is increasing in both males and females, and SKCM caused approximate 72% of deaths in skin carcinoma [1]. SKCM lacks effective treatment except early surgical resection, and it usually results in a poor prognosis and shockingly high mortality [2]. Meanwhile, SKCM is highly heterogeneous, which makes personalized treatment difficult [3]. Immune checkpoint inhibitors, such as cytotoxic T-lymphocyte antigen-4 (CTLA-4) and programmed cell death protein 1 (PD-1) inhibitors, are the main treatment measures for advanced-stage SKCM, but the efficacy of immunotherapy is limited due to different response rates [4]. In addition, primary and secondary resistance and the absence of predictive markers of response are biological challenges and clinical dilemmas in immunotherapy for SKCM patients [5]. Thus, it is urgent to identify a novel predictive biomarker for the immunotherapy of SKCM patients.

Solute carrier family 25 member 13 (SLC25A13), a calcium-binding/stimulated aspartate–glutamate carrier, plays a key role in metabolic pathways including aerobic glycolysis, gluconeogenesis, urea cycle, and synthesis of proteins and nucleotides [6]. The genetic deficiency of SLC25A13 causes adult-onset type II citrullinemia [7]. Furthermore, pathogenic germline variants correlated with increased tumor mutational burden have been found in 7 genes, including SLC25A13 and TP53, and germline variants can predict the response to immune checkpoint inhibitors [8]. In addition, SLC25A13 is correlated with tumor aggressiveness and poorer prognosis of colorectal cancer [9]. Thus, it is of importance to reveal the role of SLC25A13 in cancer development and evaluate the prognostic value of immunotherapy of SKCM patients.

**Figure 1:**
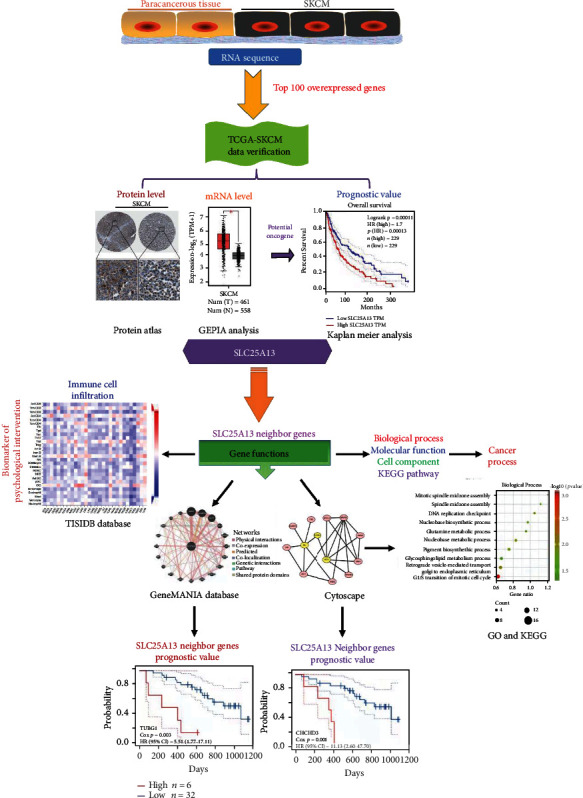
The flow chart.

This study compared the gene transcription levels between SKCM and Paracancerous tissue using RNA sequencing (data not shown), and the top 100 genes overexpressed in SKCM were considered as potential oncogenes involved in cancer process and verified by the cancer genome atlas (TCGA) data sets of SKCM. In this study, we investigated the diagnostic and prognostic value of SLC25A13 for SKCM patients and clarified the correlation between the expression of SLC25A13 and immune cell infiltration in SKCM patients; meanwhile, we also predicted the potential gene function of SLC25A13 by enrichment analysis of its neighbor genes in SKCM (Figure 1).

## 2. Methods

### 2.1. Analysis of the Expression of SLC25A13 in SKCM and Normal Skin Tissues

Gene Expression Profiling Interactive Analysis (GEPIA, http://gepia.cancer-pku.cn/) is a web-based tool, and The Cancer Genome Atlas (TCGA) and Genotype Tissue Expression (GTEx) database provide fast and customizable functionalities [10]. In this study, the expression of SLC25A13 in SKCM and normal skin tissues was compared and represented by box plots via the GEPIA. In addition, the overall survival and disease-free survival of SKCM patients based on the expression of SLC25A13 were analyzed using GEPIA.

Human Protein Atlas (https://www.proteinatlas.org) provides the expression profiles in human tissues of genes both on the mRNA and protein level [11]. The protein levels of SLC25A13 were compared in SKCM and normal skin tissues by Human Protein Atlas.

### 2.2. Survival Analyses in SKCM Patients

PROGgene (http://www.compbio.iupui.edu/proggene) serves as a tool to perform survival analysis on single genes, multiple genes as a signature, ratio of expression of two genes, and curated/published gene signatures [12]. The overall survival of SKCM patients based on the expression of SLC25A13 was analyzed using PROGgene.

PrognoScan (http://dna00.bio.kyutech.ac.jp/PrognoScan/) shows the prognostic values of genes in multiple microarray datasets with meta-analysis [13]. PrognoScan was used to explore the prognostic value of interacting genes of SLC25A13 in SKCM patients.

### 2.3. Clinical Prediction Value of the Established Prognostic Model

The TCGA database (https://portal.gdc.cancer.gov/) and the UCSC Xena database (https://xena.ucsc.edu/) were used to obtain SKCM data. All subsequent analyses were performed via *R* version 4.1.3. To test whether the expression of SLC25A13 was the effective prognostic indicator, the univariate and multivariate Cox regressions were performed [14, 15]. Then, diagnostic ROC and PR analysis of the SLC25A13 expression in normal samples and SKCM samples was performed by *R* package “ROCR” [16]. Finally, to assess the risk of death for SKCM, a nomogram was constructed by combining the expression of SLC25A13 and clinical data. The patient “TCGA-EE-A2A2-06A” was illustrated.

### 2.4. Correlation Analysis of Immune Mutation

The mutation analysis of SKCM was used the *R* package “maftools” [17, 18]. In the analysis process, a sample “TCGA-FW-A3R5-06A-11D-A23B-08 31097” was deleted because its mutation count is an outlier.

### 2.5. Correlation Analysis between the Expression of SLC25A13 and Immune Features in SKCM Patients

TISIDB (http://cis.hku.hk/TISIDB/index.php) is a website portal for interaction between tumor and immune systems in 28 types of tumor-infiltrating lymphocytes across human cancers [19]. The correlation between the abundance of tumor-infiltrating lymphocytes and the expression of SLC25A13 was investigated by TISIDB, and the relationship between abundance of immunomodulators and the expression of SLC25A13 was determined using TISIDB.

### 2.6. Analysis of Coexpressed Genes of SLC25A13 in SKCM

The multiomics data from all 32 TCGA cancer types and 10 Clinical Proteomics Tumor Analysis Consortium (CPTAC) cancer cohorts are available at LinkedOmics (http://www.linkedomics.org) [20]. In this study, LinkFinder module was used to investigate the coexpression genes of SLC25A13, and the LinkInterpreter module was employed to perform enrichment analysis of co-expression genes associated with SLC25A13 in SKCM.

The STRING database (http://string-db.org/) aims to predict protein-protein associations including direct (physical) and indirect (functional) relations from knowledge transfer between organisms and interactions aggregated from other databases [21]. Gene Ontology (GO) and KEGG analysis of SLC25A13 coexpressed genes were evaluated by the STRING database to predict the biological functional of SLC25A13.

Cytoscape is an open source software project for integrating biomolecular interaction networks with high-throughput expression data and other molecular states into a unified conceptual framework [22]. Cytoscape and MCODE were established to estimate the most indispensable module in the protein-protein interaction networks.

### 2.7. Functional Enrichment Analysis and Gene Set Enrichment Analysis (GSEA) of SLC25A13

GeneMANIA (http://www.genemania.org), a flexible web interface, is utilized for generating hypotheses about gene function, analyzing gene lists, and prioritizing genes for functional assays [23]. In the present study, the protein-protein interaction network of SLC25A13 and its interacting genes was conducted by GeneMANIA database.

Metascape (https://metascape.org/) is a web site that combines functional enrichment, interactome analysis, gene annotation, and membership search to leverage more than 40 independent knowledge bases within one integrated portal [24]. In this study, Metascape was used to demonstrate the functional enrichment analysis of SLC25A13 and its neighboring genes.

GSEA is a powerful analytical method to perform genome-wide expression profiling of two phenotypes and evaluate cumulative changes in the expression of groups of multiple genes defined based on previous biological knowledge [25]. In this study, GSEA was applied to evaluate the biological functions and signaling pathways regulated by SLC25A13.

## 3. Results

### 3.1. SLC25A13 Is Overexpressed in SKCM Compared with Normal Skin Tissues

**Figure 2:**
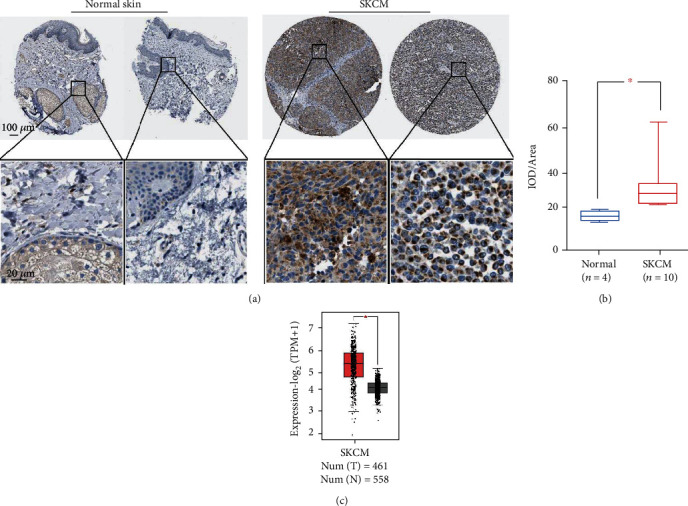
The expression of SLC25A13 in SKCM and normal skin tissues. (a) The protein levels of SLC25A13 were detected in normal skin tissues and SKCM tissues by immunohistochemistry. The data was obtained from Human Protein Atlas. × 200 magnification (bar = 100 *μ*m) and × 1000 magnification (bar = 20 *μ*m). (b) SLC25A13 was overexpressed in SKCM tissues compared with normal skin tissues. (c) The mRNA levels of SLC25A13 were analyzed by GEPIA and compared in SKCM and normal skin tissues.

Firstly, the levels of SLC25A13 in SKCM and normal skin tissues were analyzed by immunohistochemistry data collected from Human Protein Atlas [11]. As shown in Figure 2(a), high levels of SLC25A13 were detected in SKCM samples, whereas the expression of SLC25A13 could not or barely be detected in normal skin tissues. Statistical analysis manifested that the expression of SLC25A13 in the SKCM was much higher than that in normal skin tissues (Figure 2(b)). In line with the protein expression, the mRNA levels of SLC25A13 were also upregulated in SKCM compared with normal skin tissues (Figure 2(c)) [10].

### 3.2. High Levels of SLC25A13 Predict Poor Disease Outcome in SKCM

**Figure 3:**
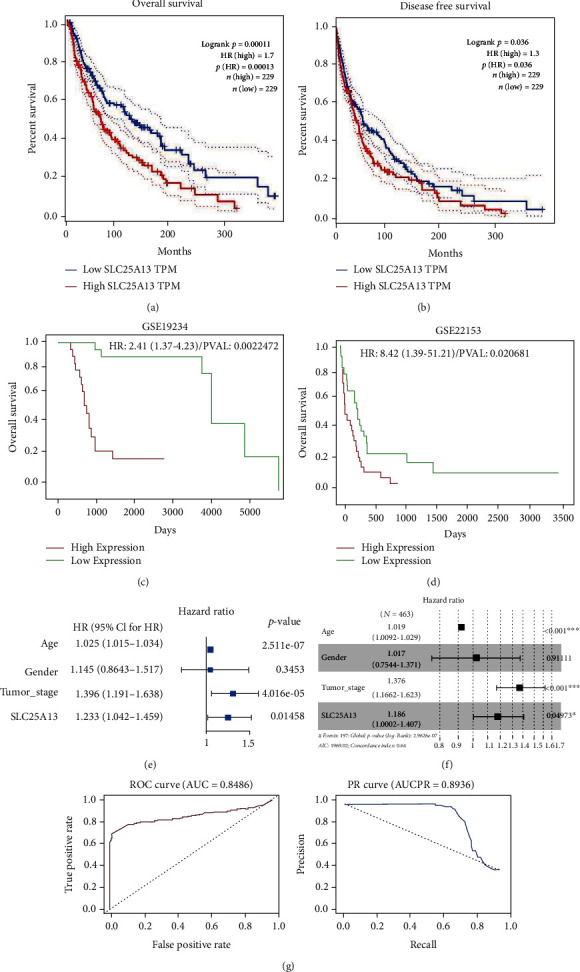
The overexpression of SLC25A13 predicts poor disease outcome in SKCM patients. (a, b) High levels of SLC25A13 predicted poor disease outcome in SKCM, and the data were obtained by GEPIA. (c, d) Overexpressed SLC25A13 predicted poor disease outcome in SKCM, and the data were obtained by PROGgene. (e) Univariate COX regression showed that SLC25A13 was an independent prognostic factor for SKCM (*p* < 0.05). (f) Multivariate COX regression showed that SLC25A13 was an independent prognostic factor for SKCM (*p* < 0.05). (g) ROC analysis showed that the area under the curve (AUC) was 0.849. Meanwhile, the area under the curve (AUC) was 0.894 according to PRC analysis. These results suggest that this gene may be a good diagnostic molecule for SKCM.

**Figure 4:**
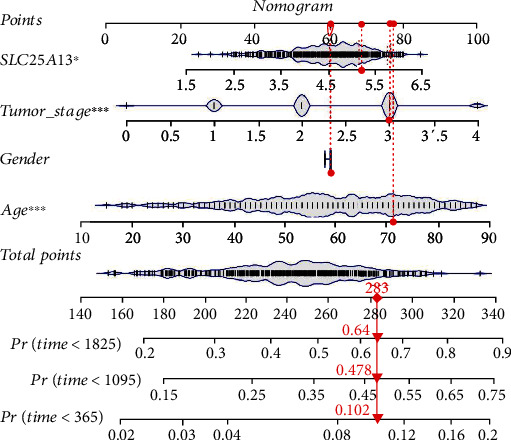
The construction of a nomogram according to the expression of SLC25A13. The 1, 3, and 5 mortality rates of patient “TCGA-EE-A2A2-06” were 0.102, 0.478, and 0.64, respectively.

To find whether the SLC25A13 expression is associated with overall survival (OS) and disease-free survival (DFS), we compared the OS and DFS of SKCM patients with high or low SLC25A13 expression. As shown in Figures 3(a) and 3(b), patients with low levels of SLC25A13 had longer OS (*p* = 0.00011) and DFS (*p* = 0.036) [10]. Furthermore, this was also validated using PROGgene based on GSE19234 and GSE22153 datasets. As shown in Figures 3(c) and 3(d), the overexpression of SLC25A13 was associated with worse OS in SKCM patients [13, 26, 27]. To explore the prognostic value of SLC25A13 gene in SKCM, univariate COX analysis was performed, and SLC25A13 was found to be an independent prognostic factor (Figure 3(e), *p* < 0.05). SLC25A13 was also found to be an independent prognostic factor by multivariate COX analysis (Figure 3(f), *p* < 0.05). To explore the diagnostic value of SLC25A13 gene in SKCM, diagnostic ROC analysis of SLC25A13 gene in 556 normal subjects and 468 SKCM patients was performed. ROC analysis showed that the area under the curve (AUC) was 0.849 (Figure 3(g)). Meanwhile, the area under the curve (AUC) was 0.894 according to PRC analysis. These results indicated that SLC25A13 might be a good diagnostic molecule for SKCM. To further determine the survival of SKCM patients, we drew a nomogram combining the risk value and clinical characteristics of the model [28]. As shown in Figure 4, we found that the 1-, 3-, and 5-year mortality rates of patient “TCGA-EE-A2A2-06” were 0.102, 0.478 and 0.64, respectively.

### 3.3. The SLC25A13 Expression Is Associated with Immune Infiltration of Tumor Immune Cells and Immunomodulators in SKCM

**Figure 5:**
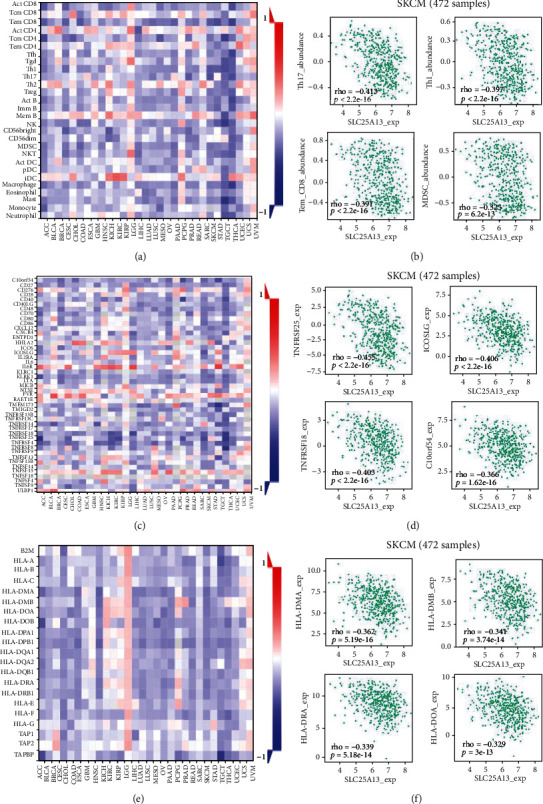
The SLC25A13 expression is linked with immune infiltration of tumor immune cells and immunomodulators in SKCM. (a, b) The TISIDB database was utilized to investigate the relations between the expression of SLC25A13 and immune infiltration of tumor immune cells. (c, d) The SLC25A13 expression was negatively correlated multiple immune-stimulators in SKCM using TISIDB. (e, f) The SLC25A13 expression was negative correlated multiple MHC molecules using TISIDB.

The TISIDB database was used to evaluate the relation between the expression of SLC25A13 and immune infiltration of tumors [19]. As shown in Figure 5(a), the level of SLC25A13 was negatively correlated with the level of 28 immune cell types in numerous cancer types. Specifically, significant negative correlations between the expression of SLC25A13 and the immune infiltration levels of multiple immune cell types in SKCM and the proportions of Th17 (*p* < 2.2*e* − 16, *R* = −0.413), Th1 (*p* < 2.2*e* − 16, *R* = −0.397), Tem CD8 (*p* < 2.2*e* − 16, *R* = −0.391), and MDSC (*p* = 6.2*e* − 13, *R* = −0.325) were lower in the high SLC25A13 expression group than in the low SLC25A13 expression group (Figure 5(b)). Besides, the SLC25A13 expression was also negatively correlated with multiple immune stimulators in SKCM (Figure 5(c)). As shown in Figure 5(d), the top-ranked genes include the following: TNFRSF25 (*p* < 2.2*e* − 16, *R* = −0.455), ICOSLG (*p* < 2.2*e* − 16, *R* = −0.406), TNFRSF18 (*p* < 2.2*e* − 16, *R* = −0.403), and C10orf54 (*p* = 1.62*e* − 16, *R* = −0.366). For the MHC molecules (Figure 5(e)), SLC25A13 was mostly negatively correlated with HLA-DMA (*p* = 5.19*e* − 16, *R* = −0.362), HLA-DMB (*p* = 3.74*e* − 14, *R* = −0.341), HLA-DRA (*p* = 5.18*e* − 14, *R* = −0.339), and HLA-DOA (*p* = 3*e* − 13, *R* = −0.329) (Figure 5(f)). These results indicated that SLC25A13 was indeed a determinant of immune infiltration and immunomodulators in SKCM.

### 3.4. Analysis of Coexpressed Genes Correlated with SLC25A13 in SKCM

**Figure 6:**
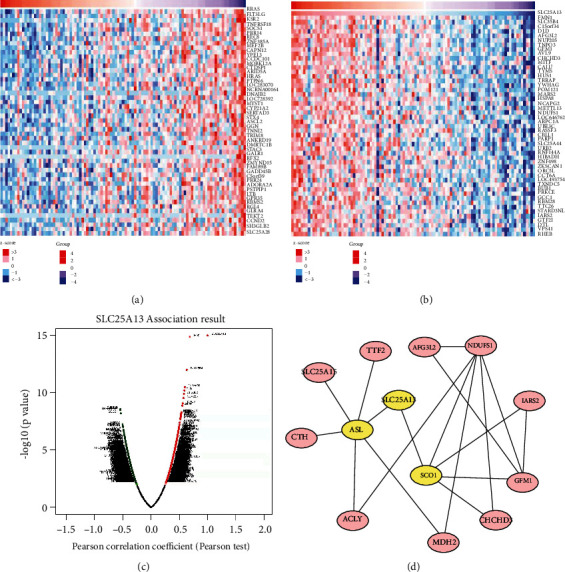
Analysis of coexpressed genes correlated with SLC25A13 in SKCM. (a, b) Heat maps showed top 50 genes positively and negatively correlated with SLC25A13. (c) The coexpressed genes of SLC25A13 were shown. (d) The important modules of SLC25A13 in the network were analyzed by Cytoscape.

To investigate the SLC25A13 coexpressed genes, we identified the potential candidate genes using LinkedOmics [20] and measured their Pearson coefficient of correlation with SLC25A13. The top 50 significant genes that were negatively (Figure 6(a)) and positively (Figure 6(b)) correlated with SLC25A13 were presented in the heat map. As shown in Figure 6(c), 8959 genes (with red label) showed statistically significant positive correlations with SLC25A13, while 10769 genes (with green label) showed statistically significant negative correlation with SLC25A13.

### 3.5. The Prognostic Value of SLC25A13 Associated Genes for SKCM Patients

**Figure 7:**
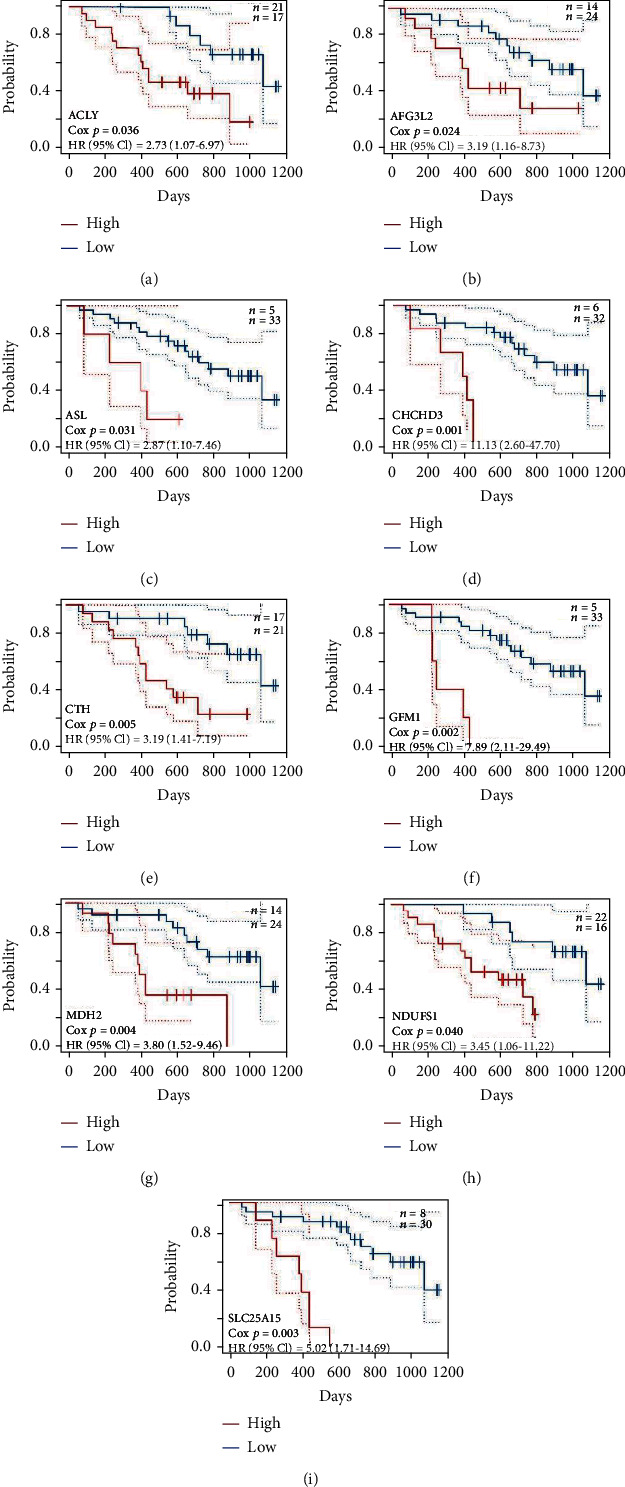
The prognosis value of SLC25A13 coexpressed genes for SKCM patients. (a)–(i) As determined by PrognoScan, high levels of SLC25A13 coexpressed genes predicted poor clinical outcome of SKCM patients, including ACLY (a), AFG3L2 (b), ASL (c), CHCHD3 (d), CTH (e), GFM1 (f), MDH2 (g), NDUFS1 (h), and SLC25A15 (i).

A total of 295 coexpressed genes with strong positive correlations with SLC25A13 were used to construct a protein-protein interaction network, and the vital module in the network was highlighted in yellow (Figure 6(d)). Furthermore, the prognostic values of SLC25A13 coexpressed genes in SKCM were investigated using PrognoScan [13]. As shown in Figures 7(a)–7(i), high level of SLC25A13 coexpressed genes predicted poor clinical outcome of SKCM patients (ACLY: hazard ratio (HR) [95%CI] = 2.73 [1.07-6.97], *p* = 0.036; AFG3L2: HR [95%CI] = 3.19 [1.16-8.73], *p* = 0.024; ASL: HR [95%CI] = 2.87 [1.10-7.46], *p* = 0.031; CHCHD3: HR [95%CI] = 11.13 [2.60-47.70], *p* = 0.001; CTH: HR [95%CI] = 3.19 [1.41-7.19], *p* = 0.005; GFM1: HR [95%CI] = 7.89 [2.11-29.49], *p* = 0.002; MDH2: HR [95%CI] = 3.80 [1.52-9.46], *p* = 0.004; NDUFS1: HR [95%CI] = 3.45 [1.06-11.22], *p* = 0.040; SLC25A15: HR [95%CI] = 5.02 [1.71-14.69], *p* = 0.003). Thus, patients with higher level of SLC25A13 and its core coexpressed genes in SKCM have poorer prognosis relative to those SKCM patients with normal SLC25A13.

**Figure 8:**
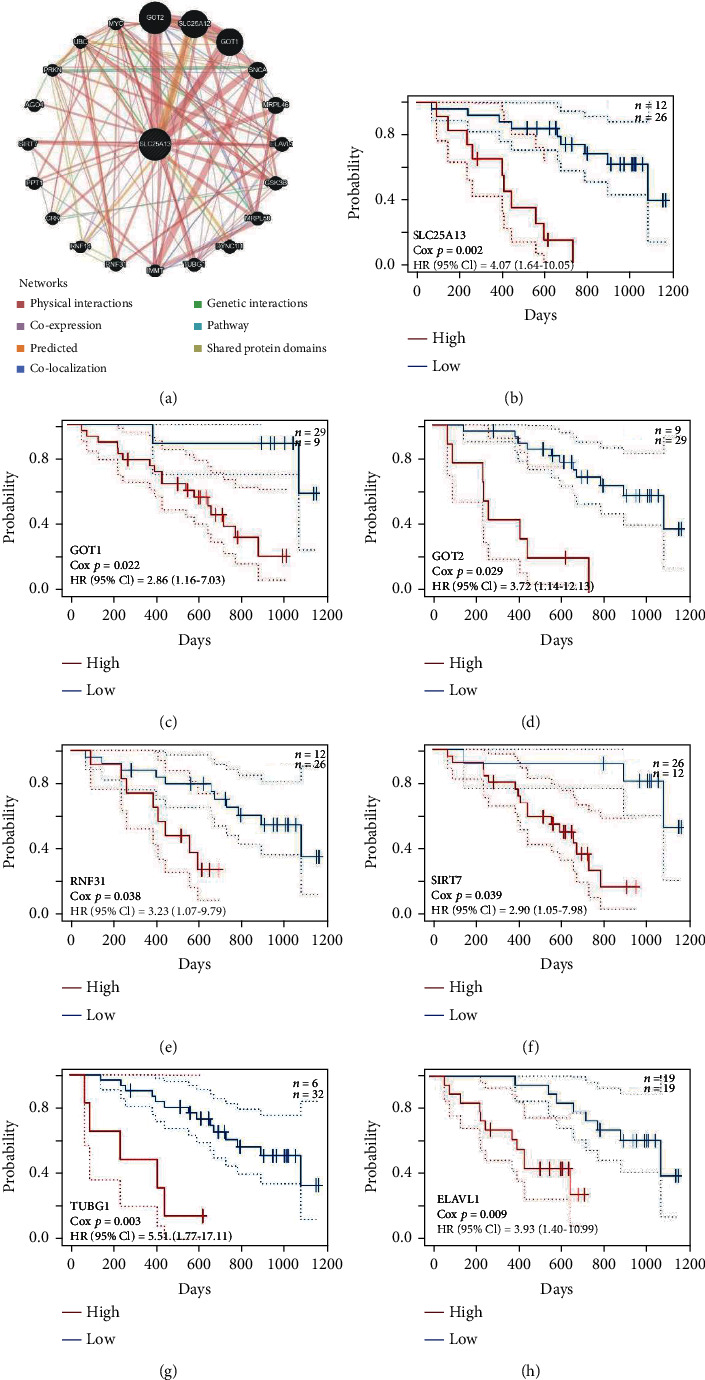
The prognosis value of SLC25A13 interacting genes for SKCM patients. (a) The protein-protein interaction network of SLC25A13 was analyzed by GeneMANIA database. (b)–(h) As determined by PrognScan, high level of SLC25A13 interacting genes predicted poor clinical outcome of SKCM patients, including SLC25A13 (b), GOT1 (c), GOT2 (d), RNF31 (e), SIRT7 (f), TUBG1 (g), and ELAVL1 (h).

To better understand the biological functions of SLC25A13, the protein-protein interaction network of SLC25A13 and its interacting genes was constructed using GeneMANIA (Figure 8(a)) [23]. Meanwhile, the prognostic values of the interacting genes of SLC25A13 were analyzed using PrognoScan [13]. As shown in Figures 8(b)–8(h), the overexpression of SLC25A13 interacting genes predicted poor clinical outcome of SKCM patients (SLC25A13: HR [95%CI] = 4.07 [1.64-10.05], *p* = 0.002; GOT1: HR [95%CI] = 2.86 [1.16-7.03], *p* = 0.022; GOT2: HR [95%CI] = 3.72 [1.14-12.13], *p* = 0.029; RNF31:HR [95%CI] = 3.23 [1.07-9.79], *p* = 0.038; SIRT7: HR [95%CI] = 2.90 [1.05-7.98], *p* = 0.039; TUBG1: HR [95%CI] = 5.51 [1.77-17.11], *p* = 0.003; ELAVL1: HR [95%CI] = 3.93 [1.40-10.99], *p* = 0.009). Altogether, the overexpression of SCL25A13 interacting genes tended to cause poor prognosis in SKCM patients.

### 3.6. GO and KEGG Analysis of SLC25A13 Coexpressed Genes

**Figure 9:**
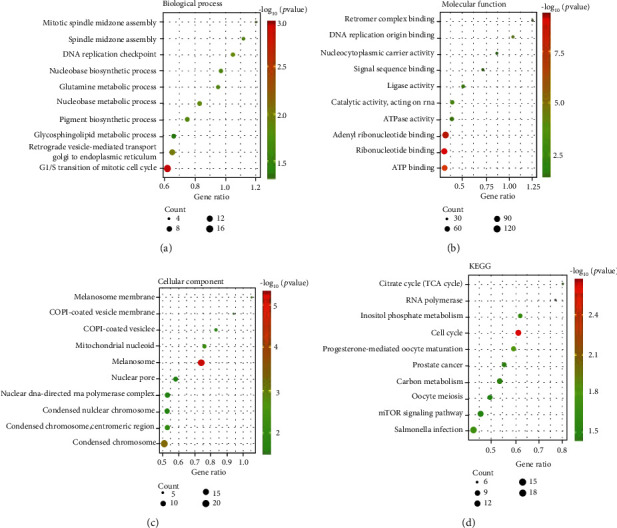
GO and KEGG pathway analysis of SLC25A13 coexpressed genes. (a) Biological process analysis of SLC25A13 coexpressed genes. (b) Molecular function analysis of SLC25A13 coexpressed genes. (c) Cell component analysis of SLC25A13 coexpressed genes. (d) KEGG pathway analysis of SLC25A13 coexpressed genes.

We input 649 coexpressed genes of SLC25A13 into the STRING database to perform functional enrichment analysis [29]. GO analysis of biological processes revealed that these genes were enriched in mitotic spindle midzone assembly, spindle midzone assembly, DNA replication checkpoint, and pigment biosynthetic process (Figure 9(a)). These genes were located in melanosome membrane, melanosome, and mitochondrial nucleoids (Figure 9(c)) and associated with the molecular functions of retromer complex binding, DNA replication origin binding, ATPase activity, etc. (Figure 9(b)). The results from KEGG analysis indicated that pathways including cell cycle and mTOR signaling pathway were particularly enriched, which were closely related to cancer progression (Figure 9(d)).

### 3.7. Functional Enrichment Analysis of SLC25A13 in Patients with SKCM

Metascape analysis showed that SLC25A13 and its neighboring genes were enriched in glucose metabolism, amino acid metabolism, and cell cycle (Supplementary Figure 1A-1B) [24]. Moreover, they were also enriched in pathways such as VEGFA-VEGFR2 signaling pathway and urea cycle and associated pathways, which were relevant to tumor development and angiogenesis. Meanwhile, SLC25A13 and its neighboring genes were enriched in transcription factor target genes, including MEF2C, NCOA2, SNAI1, and SOX10 target genes, which were participated in the cancer progression (Supplementary Figure 1 C-D).

GSEA of SLC25A13 was utilized to further investigate the biological function of SLC25A13 in SKCM by LinkedOmics [20]. GO analysis of molecular function showed that SLC25A13 positively participated in ATPase activity, while negatively regulated antigen binding and immunoglobulin binding (Supplementary Figure 2). GO analysis of the biological process showed that SLC25A13 positively participated in pigment metabolic and negatively regulated T cell activation, lymphocyte mediated immunity, humoral immune response, response to tumor necrosis factor, and leukocyte proliferation (Supplementary Figure 3). GO analysis of cell components displayed that SLC25A13 positively regulated pigment granules but negatively regulated immunological synapse and MHC protein complexes in SKCM (Supplementary Figure 4). KEGG pathway analysis showed that SLC25A13 positively regulated Fanconi anemia pathway, cell cycle, DNA replication, basal transcription factors and negatively regulated natural killer cell-mediated cytotoxicity and antigen processing and presentation (Supplementary Figure 5).

## 4. Discussion

SKCM is a highly aggressive skin tumor worldwide due its metastatic nature, and the incidence rate of SKCM keeps increasing in recent years [30]. Once SKCM spreads through the dermis, it easily spreads to vital organs and lymph nodes and causes poor prognosis [31]. Therefore, it is urgent to identify biomarkers to predict the prognosis of SKCM. This study indicated that the mRNA level and protein expression of SLC25A13 was significantly higher in SKCM than that in normal skin tissues. Moreover, Cox regression analysis showed that the SLC25A13 overexpression was an independent risk factor for poor outcome of SKCM patients. Furthermore, the overexpression of SCL25A13 associated genes including ACLY, AFG3L2, ASL, CHCHD3, CTH, GFM1, MDH2, NDUFS1, SLC25A15, GOT1, GOT2, RNF31, SIRT7, TUBG1, and ELAVL1 were also related to poor prognosis of SKCM patients. These findings revealed that SLC25A13 is a promising and prognostic biomarker in SKCM.

Multiple members of the mitochondrial carrier family (SLC25) are involved in the invasion and metastasis of many cancers. For example, Wong et al. reported that SLC25A22 promotes the proliferation and migration of colorectal cancer cells, and high levels of SLC25A22 predict poor prognosis in colorectal cancer [32]. SLC25A13 was also found to be pivotal in colorectal cancer aggressiveness [9]. Although the function of SCL25A13 in SKCM development may need further investigation, the function enrichment analysis revealed that SLC25A13 and its neighboring genes were enriched in cell cycle, VEGFA-VEGFR2 signaling, and mTOR pathway, which were related to the development of SKCM. SKCM develops from mutation of pigment-producing cells in the skin, and there is uncontrolled growth of pigment-producing cells during SKCM development [33]. In this study, the function enrichment analysis of SLC25A13 and its neighboring genes revealed that SLC25A13 was involved in pigment granules, pigment biosynthetic, and metabolic processes. Our results suggested that SLC25A13 might be critically involved in the progression of SKCM. It is well known that cancer cells have a higher metabolic rate than normal cells as a result of environmental selection and the accumulation of mutations [34]. Then, we downloaded the mutation data of SKCM through mafTools package and showed the landscape map of the top 10 genes with the highest mutation frequency, among which TTN and MUC16 genes are more prone to mutation (Supplementary Figure 6A). However, there is no relationship between SLC25A13 and tumor mutation load (TMB) through correlation analysis (Supplementary Figure 6B, COR = −0.07, *p* > 0.05). Thus, SLC25A13 was not associated with TMB of SKCM.

SLC25A13 plays a pivotal role under glucose-deprived conditions and is correlated with tumor aggressiveness [9]. The presence and amount of glucose have a great impact on the immune cell functions of the immune system, resulting in the tumor pathological stage [35]. Thus, we were interested in investigating the relationship between the expression of SLC25A13 and immune cell infiltration of SKCM. The data of the current study revealed that the SLC25A13 expression was negatively correlated with immune cell infiltration, including effector memory CD8+ and CD4+ T cells, Th1 cells, Th2 cells, and Treg cells. Furthermore, the function enrichment analysis revealed that SLC25A13 negatively regulates T cell activation, lymphocyte mediated immunity, humoral immune response, natural killer cell-mediated cytotoxicity, etc. These data indicated that the SLC25A13 expression was negatively correlated with immune cell infiltration in SKCM. Furthermore, SKCM treatment has been revolutionized with the approval of immune checkpoint inhibitors, which have a significant impact on the prognosis of patients with SKCM [36]. Nevertheless, only a small percentage of SKCM patients can benefit from immunotherapy [37]. Tumor immune cell infiltration can affect the sensitivity of immunotherapy, and higher immune cell infiltration scores showed a significant immune therapeutic advantage and clinical benefit [38]. Thus, the expression of SLC25A13 might be a potential biomarker useful for screening suitable SKCM patients for immunotherapy. Comprehensive psychological intervention can remarkably improve the negative emotions, social support degree, sleep quality, and immune functions of cancer patients receiving conventional nursing intervention during the perioperative period [39]. As SLC25A13 could be a biomarker for tumor immune cell infiltration in SKCM, the feasibility of exploring SLC25A13 as a biomarker of psychological intervention in SKCM treatment might need further investigation.

## 5. Conclusions

This work demonstrated that the SLC25A13 expression was increased in SKCM compared with normal skin tissues, and the high expression of SLC25A13 and its neighbor genes were associated with a poor prognosis in SKCM patients. In addition, the expression of SLC25A13 was negatively correlated with the tumor-infiltrating cells in the tumor immune microenvironment. Functional enrichment analysis of SLC25A13 showed that it was associated with tumor development and progression. These findings suggest that SLC25A13 may be a potential prognostic biomarker for the diagnosis and prognosis of SKCM patients.

## Data Availability

All data generated or analyzed used to support the findings of this study are included within the article and supplementary materials.

## Supplementary Materials

Supplementary Materials Supplementary Figure 1: functional enrichment analysis of SLC25A13 and its neighboring genes in patients with SKCM. (a) Heatmap of GO enriched terms stained by *p* values. (b) GO enrichment network painted by cluster ID, where nodes that share the same cluster ID are typically mutural close. (c, d) Summary of enrichment analysis in transcription factor targets. Supplementary Figure 2: GO analysis of molecular function of SLC25A13 in SKCM using LinkedOmics. (a) GSEA of SLC25A13 was utilized to further investigate the molecular function of SLC25A13 in SKCM. The correlations between SLC25A13 expression and the presence of antigen binding (b), ATPase activity (c), and immunoglobulin binding (d) were analyzed by GSEA using LinkedOmics. Supplementary Figure 3: GO analysis of biological process of SLC25A13 in SKCM using LinkedOmics. (a) GSEA of SLC25A13 was utilized to make further investigation on the biological process of SLC25A13 in SKCM. The pertinence between SLC25A13 expression and the presence of pigment metabolic (b), T cell activation (c), lymphocyte mediated immunity (d), humoral immune response (e), response to tumor necrosis factor (f), and leukocyte proliferation (g) were analyzed by GSEA using LinkedOmics. Supplementary Figure 4: GO analysis of cell component of SLC25A13 in SKCM using LinkedOmics. (a) GSEA of SLC25A13 was utilized to further investigate the cell component of SLC25A13 in SKCM. The correlations between the SLC25A13 expression and the presence of pigment granule (b), immunological synapse (c), and MHC protein complex (d) were analyzed by GSEA using LinkedOmics. Supplementary Figure 5: KEGG pathways analysis of SLC25A13 in SKCM using LinkedOmics. (a) GSEA of SLC25A13 was utilized to further investigate KEGG pathways of SLC25A13 in SKCM. The correlations between SLC25A13 expression and the presence of fanconi anemia pathway (b), cell cycle (c), basal transcription factors (d), natural killer cell mediated cytotoxicity (e), DNA replication (f), and antigen processing (g) were analyzed by GSEA using LinkedOmics. Supplementary Figure 6: mutation analysis of SKCM. (a) The landscape map of the top 10 genes with the highest mutation frequency, among which TTN and MUC16 genes are more prone to mutation. (b) SLC25A13 was not associated with tumor mutation load (TMB). (COR = −0.07, *p* > 0.05).
