# Supplementary material for: The Overexpression of SLC25A13 Predicts Poor Prognosis and Is Correlated with Immune Cell Infiltration in Patients with Skin Cutaneous Melanoma
Source: Dis Markers. 2022 May 14;2022:4091978. doi: 10.1155/2022/4091978 (PMC9124094; doi:10.1155/2022/4091978)
Supplement: Supplementary Materials — Supplementary Figure 1: functional enrichment analysis of SLC25A13 and its neighboring genes in patients with SKCM. (a) Heatmap of GO enriched terms stained by p values. (b) GO enrichment network painted by cluster ID, where nodes that share the same cluster ID are typically mutural close. (c, d) Summary of enrichment analysis in transcription factor targets. Supplementary Figure 2: GO analysis of molecular function of SLC25A13 in SKCM using LinkedOmics. (a) GSEA of SLC25A13 was utilized to further investigate the molecular function of SLC25A13 in SKCM. The correlations between SLC25A13 expression and the presence of antigen binding (b), ATPase activity (c), and immunoglobulin binding (d) were analyzed by GSEA using LinkedOmics. Supplementary Figure 3: GO analysis of biological process of SLC25A13 in SKCM using LinkedOmics. (a) GSEA of SLC25A13 was utilized to make further investigation on the biological process of SLC25A13 in SKCM. The pertinence between SLC25A13 expression and the presence of pigment metabolic (b), T cell activation (c), lymphocyte mediated immunity (d), humoral immune response (e), response to tumor necrosis factor (f), and leukocyte proliferation (g) were analyzed by GSEA using LinkedOmics. Supplementary Figure 4: GO analysis of cell component of SLC25A13 in SKCM using LinkedOmics. (a) GSEA of SLC25A13 was utilized to further investigate the cell component of SLC25A13 in SKCM. The correlations between the SLC25A13 expression and the presence of pigment granule (b), immunological synapse (c), and MHC protein complex (d) were analyzed by GSEA using LinkedOmics. Supplementary Figure 5: KEGG pathways analysis of SLC25A13 in SKCM using LinkedOmics. (a) GSEA of SLC25A13 was utilized to further investigate KEGG pathways of SLC25A13 in SKCM. The correlations between SLC25A13 expression and the presence of fanconi anemia pathway (b), cell cycle (c), basal transcription factors (d), natural killer cell mediated cytotoxicity (e), DNA replication (f), and a [file 4091978.f1.docx]

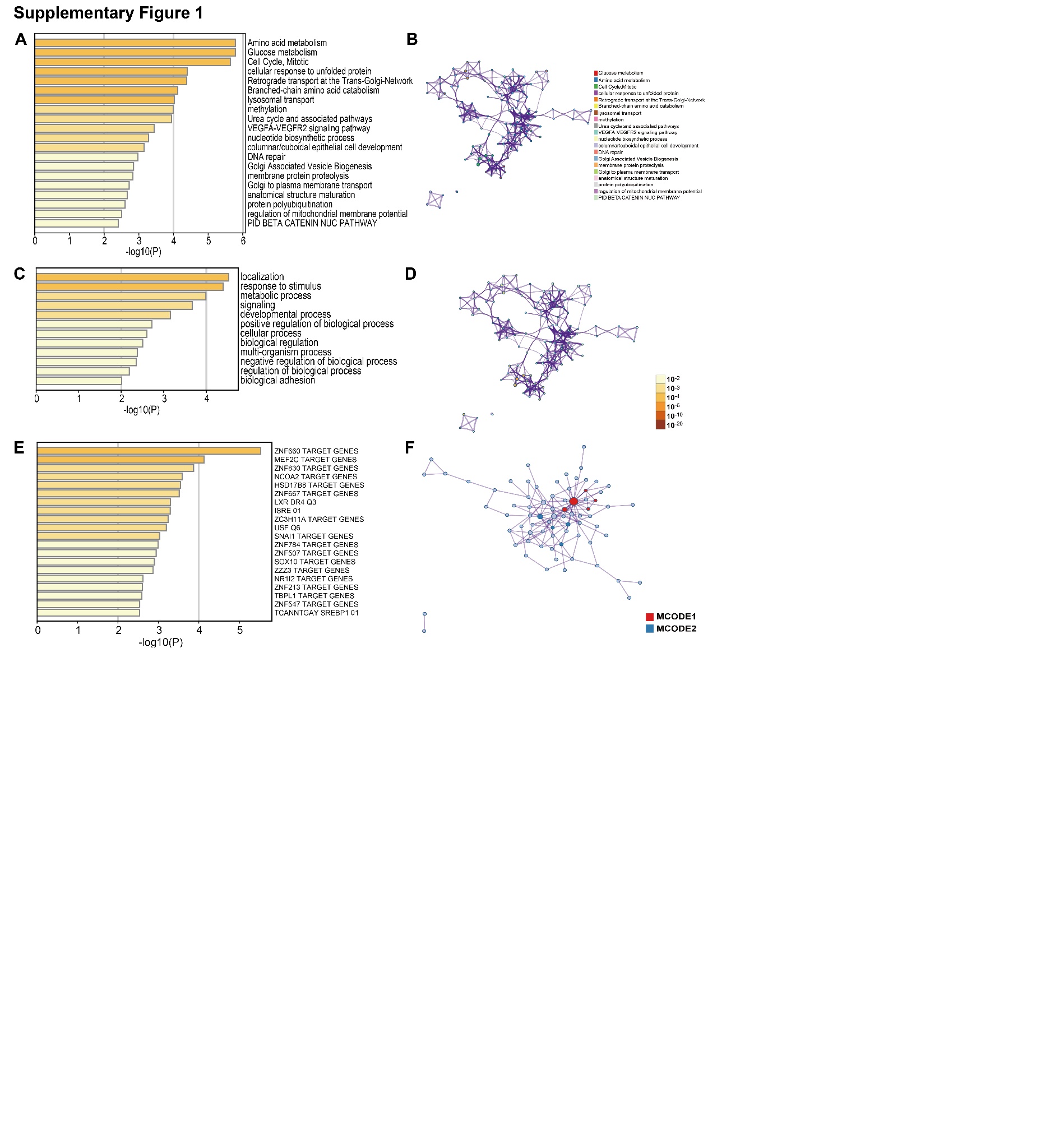
**Supplementary Figure 1: Functional enrichment analysis of SLC25A13 and its neighboring genes in patients with SKCM**. (A) Heatmap of GO enriched terms stained by p values. (B) GO enrichment network painted by cluster ID, where nodes that share the same cluster ID are typically mutural close. (C-D) Summary of enrichment analysis in transcription factor targets.


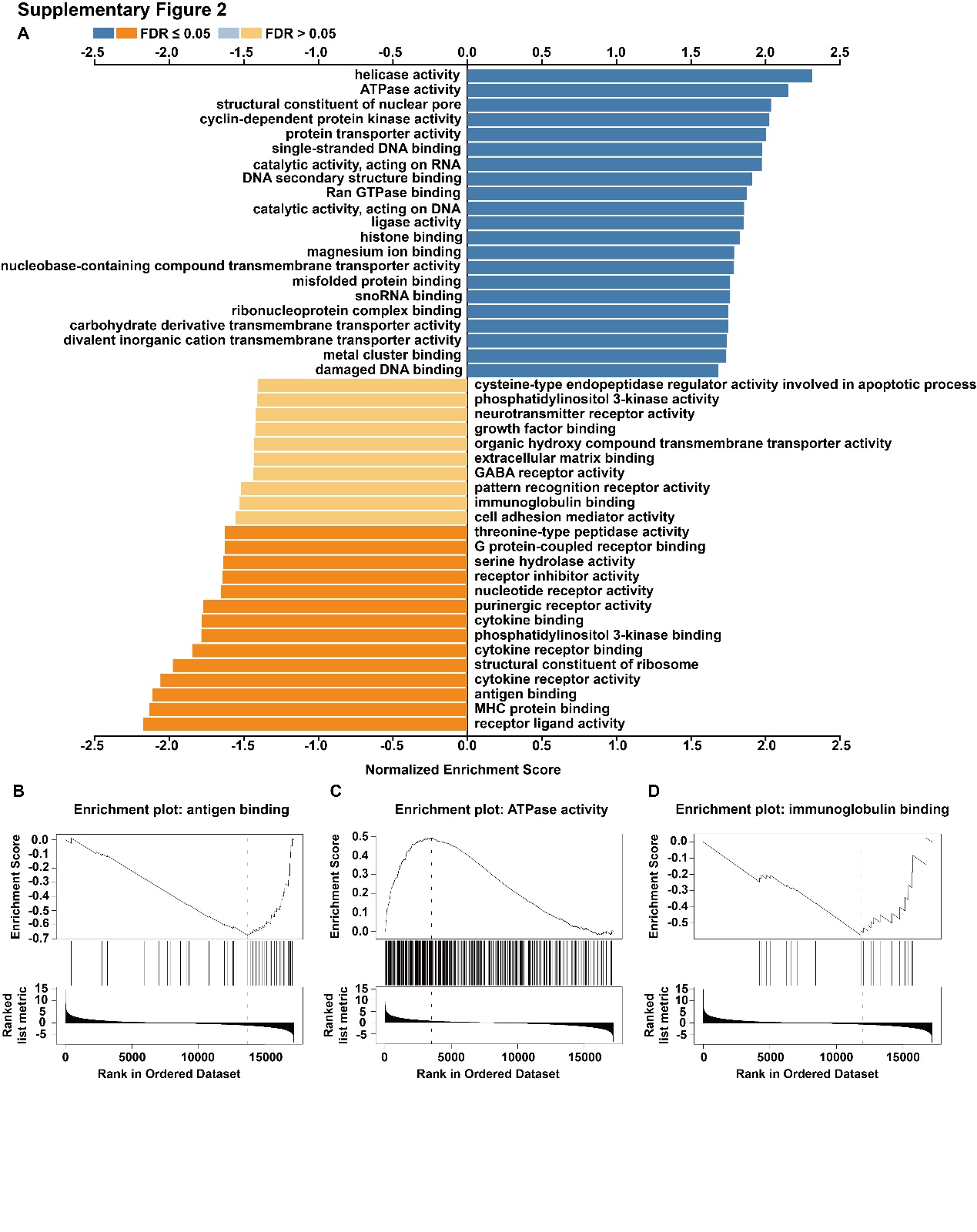


**Supplementary Figure 2: GO analysis of molecular function of SLC25A13 in SKCM using LinkedOmics.** (A) GSEA of SLC25A13 was utilized to further investigate the molecular function of SLC25A13 in SKCM. The correlations between SLC25A13 expression and the presence of antigen binding (B), ATPase activity (C), and immunoglobulin binding (D) were analyzed by GSEA using LinkedOmics.
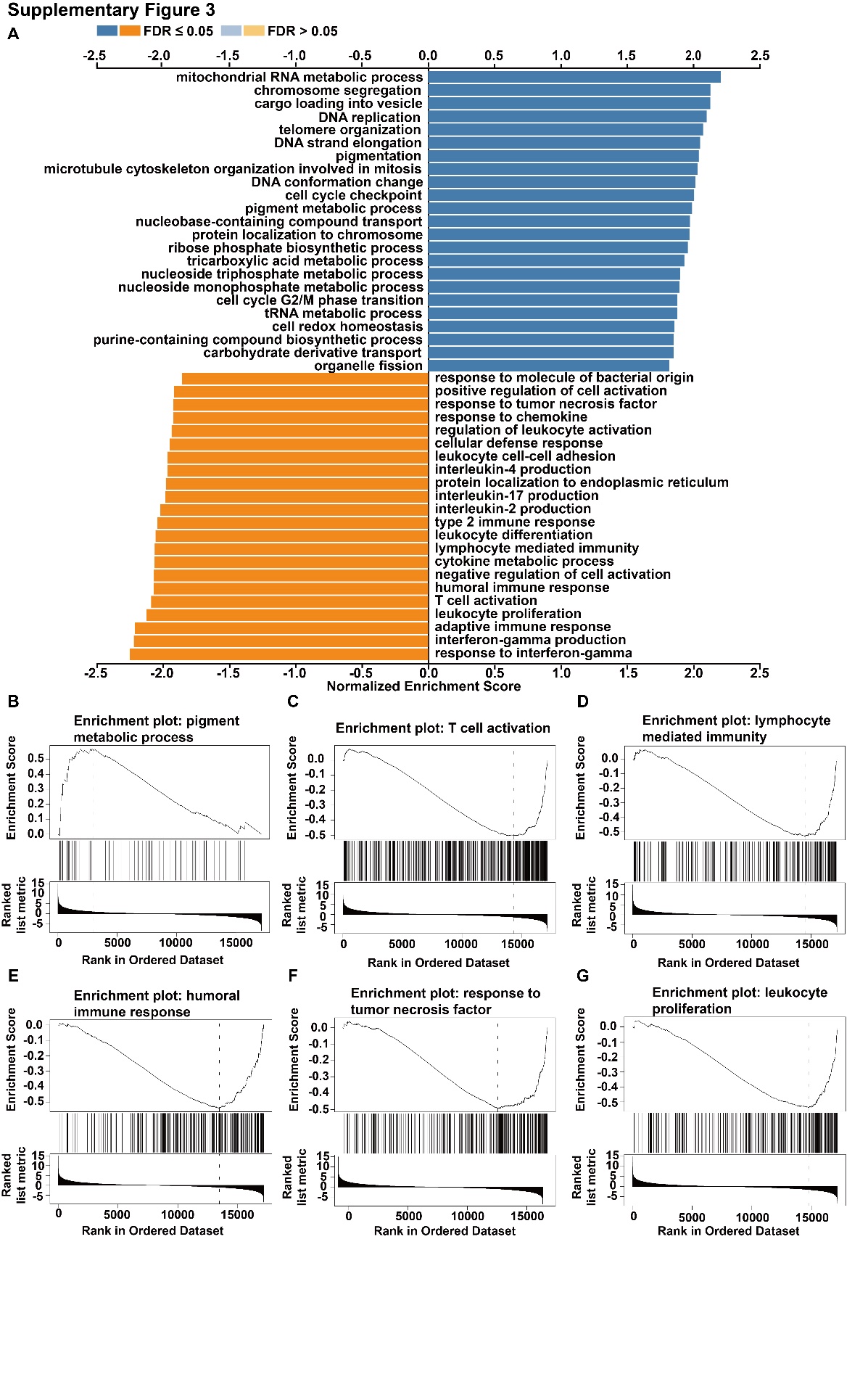


**Supplementary Figure 3: GO analysis of biological process of SLC25A13 in SKCM using LinkedOmics.** (A) GSEA of SLC25A13 was utilized to make further investigation on the biological process of SLC25A13 in SKCM. The pertinence between SLC25A13 expression and the presence of pigment metabolic (B), T cell activation (C), lymphocyte mediated immunity (D), humoral immune response (E), response to tumor necrosis factor (F), leukocyte proliferation (G) were analyzed by GSEA using LinkedOmics.


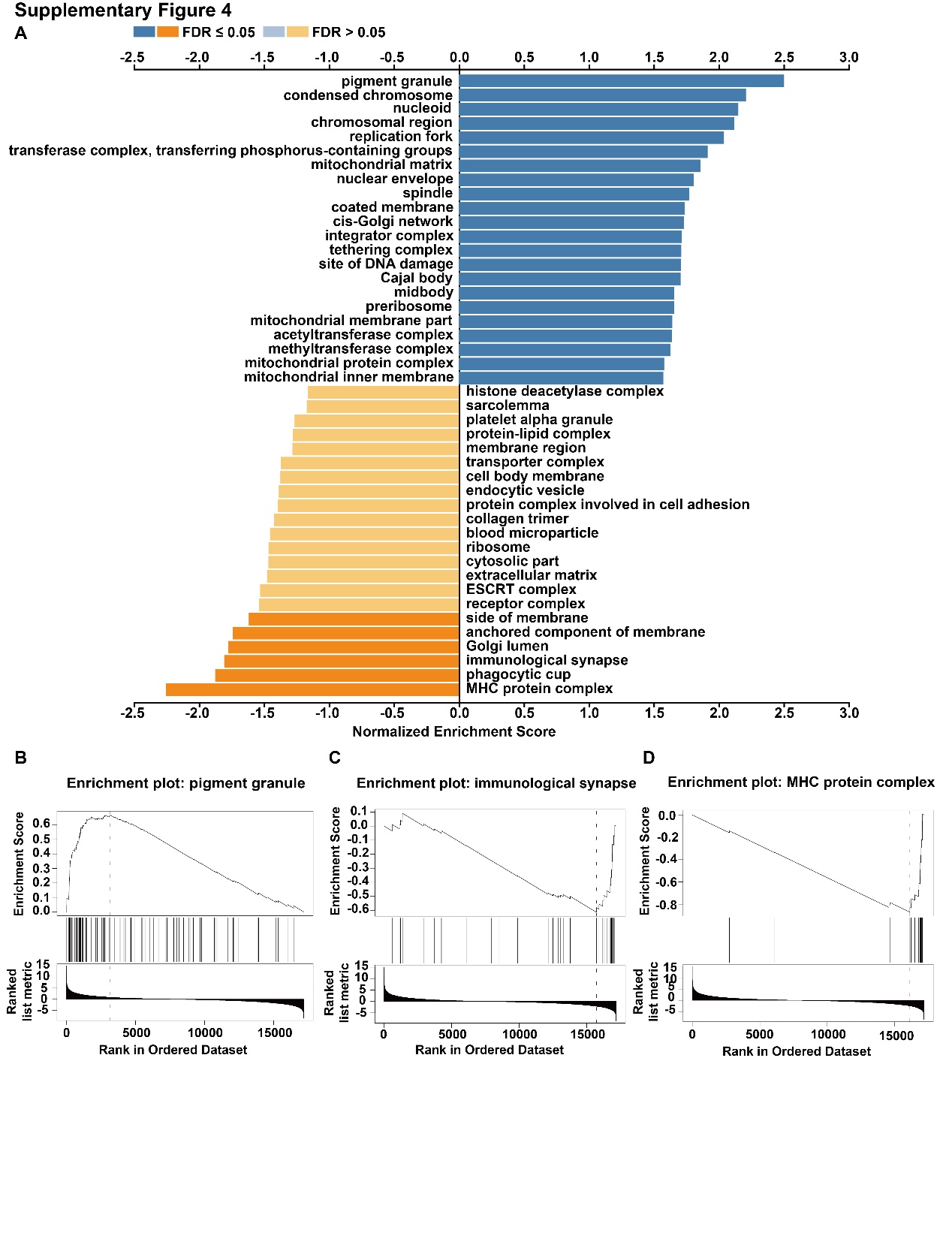


**Supplementary Figure 4: GO analysis of cell component of SLC25A13 in SKCM using LinkedOmics.** (A) GSEA of SLC25A13 was utilized to further investigate the cell component of SLC25A13 in SKCM. The correlations between SLC25A13 expression and the presence of pigment granule (B), immunological synapse (C), and MHC protein complex (D) were analyzed by GSEA using LinkedOmics.


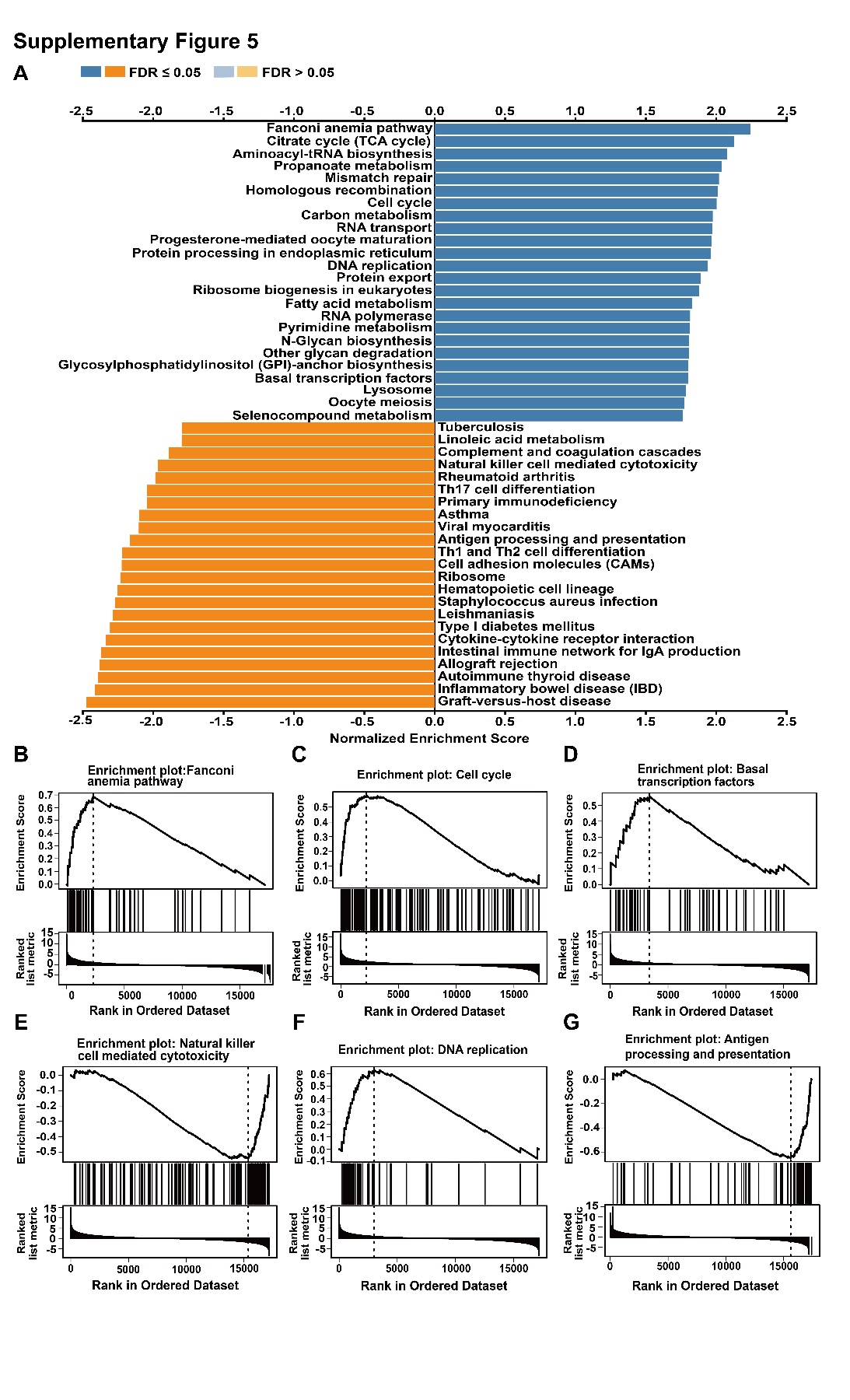


**Supplementary Figure 5: KEGG pathways analysis of SLC25A13 in SKCM using LinkedOmics.** (A) GSEA of SLC25A13 was utilized to further investigate KEGG pathways of SLC25A13 in SKCM. The correlations between SLC25A13 expression and the presence of fanconi anemia pathway (B), cell cycle (C), basal transcription factors (D), natural killer cell mediated cytotoxicity (E), DNA replication (F), and antigen processing (G) were analyzed by GSEA using LinkedOmics.

**
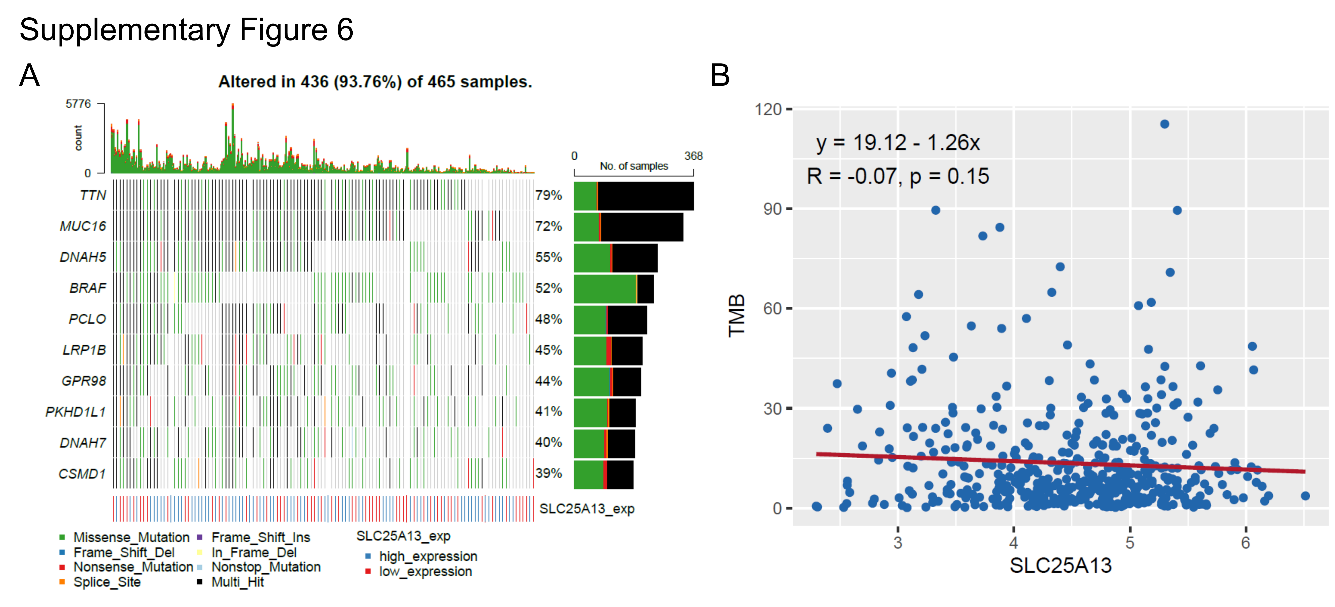
Supplementary Figure 6: Mutation analysis of SKCM.** (A) The landscape map of the top 10 genes with the highest mutation frequency, among which TTN and MUC16 genes are more prone to mutation. (B) SLC25A13 was not associated with tumor mutation load (TMB). (COR = -0.07, P >0.05).
